# G1-phase progression in pluripotent stem cells

**DOI:** 10.1007/s00018-021-03797-8

**Published:** 2021-04-21

**Authors:** Menno ter Huurne, Hendrik G. Stunnenberg

**Affiliations:** 1 Department of Molecular Biology, Faculty of Science, Radboud University, 6525GA Nijmegen, The Netherlands; 2 Present Address: Murdoch Children’s Research Institute, Royal Children’s Hospital, Flemington Rd, Parkville, Melbourne, VIC 3052 Australia; 3 Present Address: Princess Maxima Centre for Pediatric Oncology, Heidelberglaan 25, 3584 CS Utrecht, The Netherlands

**Keywords:** Cell Cycle, Pluripotency, G1-phase, Embryonic stem cells

## Abstract

During early embryonic development both the rapid increase in cell number and the expression of genes that control developmental decisions are tightly regulated. Accumulating evidence has indicated that these two seemingly independent processes are mechanistically intertwined. The picture that emerges from studies on the cell cycle of embryonic stem cells is one in which proteins that promote cell cycle progression prevent differentiation and vice versa. Here, we review which transcription factors and signalling pathways play a role in both maintenance of pluripotency as well as cell cycle progression. We will not only describe the mechanism behind their function but also discuss the role of these regulators in different states of mouse pluripotency. Finally, we elaborate on how canonical cell cycle regulators impact on the molecular networks that control the maintenance of pluripotency and lineage specification.

## Introduction

Embryonic stem cells (ESCs) are the subject of intense research. These cells derived from the inner cell mass (ICM) of the early blastocyst can be propagated indefinitely in vitro while retaining the transcriptional and epigenetic properties of their in vivo counterparts. As a result, these in vitro cultured cells maintain the ability differentiate into all cells of the fully developed body plan (termed “pluripotent”). Unlimited in vitro expansion allows scaling necessary for molecular analysis and has facilitated unraveling the molecular mechanisms that dictate maintenance of pluripotency and differentiation. The transcriptional network that ensures the pluripotent phenotype of embryonic stem cells is at the core controlled by three Transcription Factors (TFs), OCT4, SOX2 and NANOG. These TFs have overlapping genomic binding sites and uphold a transcriptional network that favors the expression of pluripotency genes and inhibits lineage specifying genes [1]. Cell type specification on the other hand is driven by lineage-specific signaling pathways and TFs that regulate epigenetic modifications that alter the cells’ genomic structure and transcriptional program [2, 3]. This knowledge has allowed scientists to recapitulate these processes in vitro and to develop a wide range of protocols that can be used to create specific cell types and even fully developed and functional organs in vitro [4]. These methods not only hold great promise to replace lost tissue but have also opened new exciting opportunities to study development and disease [5].

The identification of pathways that initiate differentiation has also resulted in distinct culture methods that allow self-renew and maintenance of pluripotency. Up to almost one decade ago mouse ESCs were cultured in conditions that included serum components or Bone Morphogenetic Proteins (BMPs) to prevent differentiation. The realization that the BMP acts through the inhibition of fibroblast growth factor (FGF)/extracellular signal-regulated kinase (ERK) signaling paved the way for the development of a serum-free condition that includes two small molecule inhibitors (2i) to support in vitro propagation of pluripotent mouse ESCs [6]. Transcriptional and epigenetic profiling implied that serum ESC populations are naive pluripotent cells (hereafter referred to as “naive ESCs”) but rather heterogeneous in terms of morphology and the expression of core pluripotency genes. ESCs grown in 2i conditions (hereafter referred to as “ground state ESCs”) on the other hand are more homogeneous and are considered to “occupy a ground state in which the pluripotency gene regulatory circuitry is maximally operative” (reviewed by [7]).

During embryonic development lineage specification is paralleled by a steady increase in cell number. In between mitotic cell division proliferating cells pass consecutively through Gap1 (G1)-phase, Synthesis (S)- phase and G2-phase. Pluripotent cells from the ICM are characterized by extremely short gap-phases that lengthen upon lineage specification during the time of implantation (reviewed by [8]). Studies on the 3D chromatin conformation revealed that cell type-specific chromatin configurations are detected most prominently during G1-phase and that genomic interactions that drive gene expression, like enhancer-promoter interactions, are taking place during G1-phase [9]. Similarly, in ESCs the expression of lineage specifiers as well as the establishment of bivalent domains at the promoters of developmental genes occurs specifically during G1-phase and are accompanied by chromatin reorganization and enhancer-promoter interactions [10, 11]. The recently discovered cell cycle phase-specific activity of the Polycomb complexes could be one of the processes that contribute to this phenomenon [12]. Together, these findings have led scientists to believe that the G1-phase serves as an window of opportunity for ESCs to initiate differentiation (reviewed by [13]). In line with this hypothesis, several studies have indicated that mouse and human ESCs are more sensitive to differentiation-inducing conditions in G1-phase when compared to either S- or G2-phase and that cells residing in G1-phase show reduced colony-forming capacities [14, 15].

The G1-phase of cells both from the ICM as well as from in vitro cultured naive mouse ESCs is extremely short. If differentiation associated with chromatin remodeling is indeed specifically initiated during G1-phase this characteristic short G1-phase of ESCs could potentially serve as a barrier to prevent differentiation. Although elongation of G1-phase correlates well with differentiation and delaying S-phase entry has been shown to induce differentiation in some cases [16, 17] fast progression through the G1-phase is not a prerequisite for ESCs to maintain pluripotent. The most compelling evidence for this is in vivo diapause. During a period that in mice can take up to 20 days, the transcriptional activity of the pluripotent cells in blastocyst is severely reduced and there is minimal cell division [18]. In vitro diapause can be recapitulated by inhibiting either MYC or the mTOR pathway [19, 20]. Although the former stalls ground state ESCs in G1-phase it does not affect their pluripotent potential [19]. In line with these observations changes in the distribution of cells over the different phases of the cell cycle upon the adaptation of ESCs to ground state conditions imply that the extremely short G1-phase as observed in naive ESCs is not an intrinsic property of ESCs but is at least in part the result of abundant external stimuli [21–23]. In spite of these differences in G1-phase, cells in both states of pluripotency proliferate at roughly the same pace implying that rapid proliferation is a characteristic feature of ESCs. What causes these cells to proliferate at such a rapid pace is still not entirely clear.

Accumulating evidence indicates that signaling pathways and transcriptions factors of the core pluripotency network impact the cell cycle. In this communication, we will review how the pluripotency network impacts on the cell cycle, mainly focusing on OCT4, SOX2, and NANOG. Most of the studies on cell cycle regulation in ESCs so far have been performed, however, using naive ESCs. Although the number of reports on cell cycle control in ground state ESCs is still limited they have revealed some striking differences regarding the cell cycle when compared to naive ESCs. Throughout the review, we will also discuss these differences and elaborate on their implications. Finally, we will discuss how cell cycle regulators modulate pluripotency and differentiation.

### Cell cycle control in different states of pluripotency

The characteristic of classically cultured naive ESCs is that they lack the control mechanisms that prevent S-phase entry. The canonical pathway that regulates G1-phase progression culminates in E2F activity that drives progression into S-phase. The E2F transcription factors are inhibited by the pocket protein family—consisting of RB, P107, and P130- that in turn are inhibited by CDK/cyclin-mediated phosphorylation. Two families of CDK-inhibitors exist, the CIP/KIP and the INK4/ARF that can inhibit CDK/cyclin complex formation. One of the first observations that explained the characteristic fast ESC cell cycle was the absence of hypo-phosphorylated RB in naive ESCs [24]. Subsequent studies revealed that the RB pathway is compromised in these cells and not activated in growth-inhibitory conditions (reviewed by [25]. Several mechanisms might act in concert to prevent the expression of hypo-phosphorylated RB.

One possible explanation is that in naive ESCs not only the relative amount of hypo-phosphorylated active RB but also the total RB protein level is low in naive ESCs compared to Mouse Embryonic Fibroblasts (MEFs) [24]. Low RB levels imply that minimal CDK/cyclin activity suffices to induce S-phase entry and could explain rapid proliferation despite the low expression of CDKs in ESCs [26]. The low expression of these pocket proteins is amongst others the result of the expression of an ESC-specific set of microRNAs that targets its mRNA [27]. Besides a low basal pocket protein expression level, the short G1-phase and the inability of ESCs to arrest in G1-phase upon stress has also been attributed to precocious CDK/Cyclin activity resulting in hyper-phosphorylation and inactivation of RB. In contrast to somatic cells ESCs display a non-cyclic expression of the cyclins and early studies have indicated that fast progression through G1-phase in naive ESCs is mediated by constitutive CDK/cyclin expression and the absence of CDK-inhibitors [28]. Naive ESCs in general do not express CDK-inhibitors and in part seem insensitive to the ectopic expression [26, 29, 30]. It is not entirely clear why CDKi’s are not expressed in naive ESCs but both the activity of microRNAs as well as nonsense-mediated decay are likely to contribute to the repression of the CIP/KIP family of CDK-inhibitors [31, 32].

Although this characteristic ESC-specific cell cycle is fundamentally different from the somatic cell cycle the exact molecular mechanism underlying remained elusive for more than three decades. Below we will discuss how certain signaling pathways and TFs of the core pluripotency network impact on the cell cycle of naive and ground state ESCs.

**Table:** 

Pathways/TF	Target*	Function	References
FGF/ERK	Ccnd	Promotes G1-phase progression	[34, 36]
Wnt	Ccnd, Ccne	Promote G1-phase progression	[38]
	Ink4a/Arf	Inhibits G1-phase progression	[39]
Oct4	LincRNA	Promotes G1-phase progression	[41]
	MicroRNA	Promotes G1-phase progression	[27, 42]
Sox2	Cyclins	Promotes G1-phase progression	[46–48]
	Cdkn1a	Promotes G1-phase progression	[49, 50]
Nanog	Cdk6	Promotes G1-phase progression	[52]
	Cdc25	Promotes G1-phase progression	[51]
	Cdk1b	Promotes G1-phase progression	[53]
Myc	Ccnd	Promotes G1-phase progression	[58]
	MicroRNA	Promotes G1-phase progression	[59]

*Targets negatively or positively affected by the Pathway/TF are color-coded in red or green, respectively

### FGF/ERK and WNT signaling

The FGF/ERK-pathway is involved in primitive endoderm formation and germline specification in the early embryo and its inhibition allows to maintain ESCs in ground state pluripotency [6, 33]. Activation of the ERK pathway results in the elevated activity of CDK/cyclin complexes and drives progression through G1-phase (reviewed in [34]). We and others have recently shown that ERK-signaling plays major role in phosphorylation of RB and S-phase entry in naive mouse ESCs [22, 35]. How ERK exactly controls phosphorylation is not entirely clear although it is likely that it involves the expression of CYCLIN D [22, 36]. In ground state, ESCs FGF/ERK signaling is inhibited leading to lowered expression of Cyclin D and more cells in the G1-phase [22]. The fact that inhibition of ERK signaling in naive ESCs did not fully restore the G1-checkpoint as present in ground state ESCs suggests that other processes contribute to the shortening of G1-phase in naive ESCs [22].

Besides ERK signaling, the activity of cell cycle regulators is also affected by Wnt signaling which is a crucial mediator of pluripotency [37]. Positive regulation of both Cyclin D and Cyclin E is mediated via direct transcriptional control by β-catenin as well as through inhibition of GSK3, which targets both cyclins for degradation. Wnt signaling thereby is an important positive regulator of G1- to S-phase progression in somatic cells (reviewed by [38]). In contrast to somatic cells, active Wnt signaling has been shown to reduce the speed with which ground ESCs progress through G1-phase [39]. Upon active Wnt signaling one of the two major downstream TFs, TCF1, was recruited to the INK4/Arf tumor suppressor locus and induced the expression of the CDKi’s P16 and P19. Corollary S-phase entry was delayed and the proliferation of naive ESCs was slowed down without affecting their undifferentiated state.

The differences in cell cycle between naive and ground state ESCs are therefor at least in part the result of lowered CYCLIN D and elevated CDKi expression mediated by FGF/ERK inhibition and stimulation of Wnt signaling, respectively. The addition of the 2i inhibitors to naive ESCs had however a less pronounced effect on the cell cycle when compared to the adaptation to ground state serum-free conditions [22]. In ground state ESCs two members of the CIP/KIP family of CDKi’s, P21 and P27, are higher expressed when compared to naive ESCs. The expression of P21 is mediated P53 activity in ground state ESCs [40]. On the contrary, in naive ESCs the P53/P21 pathway is inactive, ensuring an extremely short G1 phase. Notably, like ground state mouse ESCs human ESCs do express CDKi’s, despite the fact that they display a short G1-phase. Several other ESC-specific mechanisms have, however, shown to contribute to the shortened G1-phase in naive ESCs. In the next section we will consider these mechanisms and elaborate on their influence on the cell cycle of naive and ground state ESCs.

### OCT4/SOX2/NANOG

Several lines of evidence have indicated that members of the pluripotency network employ multiple mechanisms to modulate progression through the G1-phase. Firstly, both OCT4 and SOX2 directly control the expression of long intergenic noncoding RNAs (lincRNAs) [41] and ESC-specific microRNAs that inhibit key regulators of G1-phase progression and thereby contribute to the abbreviated G1-phase [27, 42]. The microRNAs repress the expression of the pocket proteins as well as CDKi’s and desensitize ESCs to serum starvation [43]. Moreover, certain microRNA clusters promote ERK signalling in ESCs and thereby stimulate cell cycle progression [44]. Whether a similar mechanism is employed by OCT4 and SOX2 to repress the expression of the pocket proteins in ground state ESCs remain to be determined. However, a recent study has indicated that self-renewal of ground state ESCs has a higher dependence on microRNA than naive ESCs, which could be explained by the fact that ground state but not naive ESCs express both CDKi’s as well hypo-phosphorylated RB [45].

Secondly, CDK/Cyclin-mediated phosphorylation of RB is promoted by both SOX2 and NANOG. SOX2 can stimulate cell proliferation either directly as a transcriptional activator of several cyclins [46–48] or as a repressor of P21 [49, 50]. NANOG contributes to accelerated S-phase entry through the transcriptional activation of CDK6 and CDC25A [51, 52]. Similar to SOX2, NANOG is able to suppress the expression of CDKi’s, NANOG binds upstream of the Cdkn1b gene and its expression correlates with repression of P27 [53]. Interestingly, two independent OCT4-driven pathways mediate RB hyper-phosphorylation and G1-phase progression in naive ESCs [54, 55]. Altogether, these results imply that the pluripotency network contributes to the phosphorylation of the pocket proteins and abrogation of the G1/S checkpoint. Despite the fact that OCT4, SOX2, and NANOG are expressed at similar levels in naive and ground state ESCs the levels of CDKi’s were significantly increased and no hyper-phosphorylated RB was observed during the G1-phase of ground state ESCs, suggesting that the core pluripotency TFs do not facilitate G1-phase progression in ground state ESCs. Knockdown studies on these TFs combined with a more comprehensive analysis of the expression and phosphorylation levels of the pocket proteins in ground state ESCs are needed to determine whether the core pluripotency TFs do contribute to G1-phase progression in ground state ESCs.

### MYC

Although MYC is not considered to be part of the core pluripotency network, the MYC family of TFs has a well-established role in stem cell maintenance as well as cell cycle regulation (reviewed by [56]). MYC is a direct transcriptional regulator of both CYCLIN D and E and the MYC-mediated activation of CDK2/Cyclin E complexes has shown to overcome the restriction point is naive ESCs [57]. Although MYC is only lowly expressed in ground state ESCs when compared to naive ESCs no major differences in CYCLIN D and E expression were observed [6, 22]. MYC, however, also antagonizes the expression of P21 and the decreased expression of MYC might therefore (next to inhibition of ERK and Wnt activation) contribute to the reinstatement of G1-phase control in ground state ESCs [22, 58]. Interestingly, MYC is necessary for ground state ESCs to proliferate and its loss results in G1-phase arrest in ground state ESCs but not in naive ESCs ([19] and unpublished observations). Although MYC is well known for its role as a transcriptional activator of G1-cyclins the absence of hyper-phoshorylated pocket proteins in ground state ESCs during the late G1 phase suggests that MYC-mediated S-phase entry is not CDK/cyclin-dependent. Previous studies have shown that MYC also mediates the expression of a microRNA cluster that targets the RB family protein P130 and thereby contributes positively to cell cycle progression [59]. It would therefore be interesting to assess the expression of hypo-phosphorylated pocket proteins in ground state ESCs upon MYC-deprivation. Such studies could provide valuable insights into the role of MYC in the ESC cell cycle. The fact that inhibition of MYC does not result in G1-arrest in naive ESCs confirms that not MYC but CDK/cyclin-driven activation of E2Fs initiates S-phase entry in naive ESCs [6, 22].

**Fig. 1:**
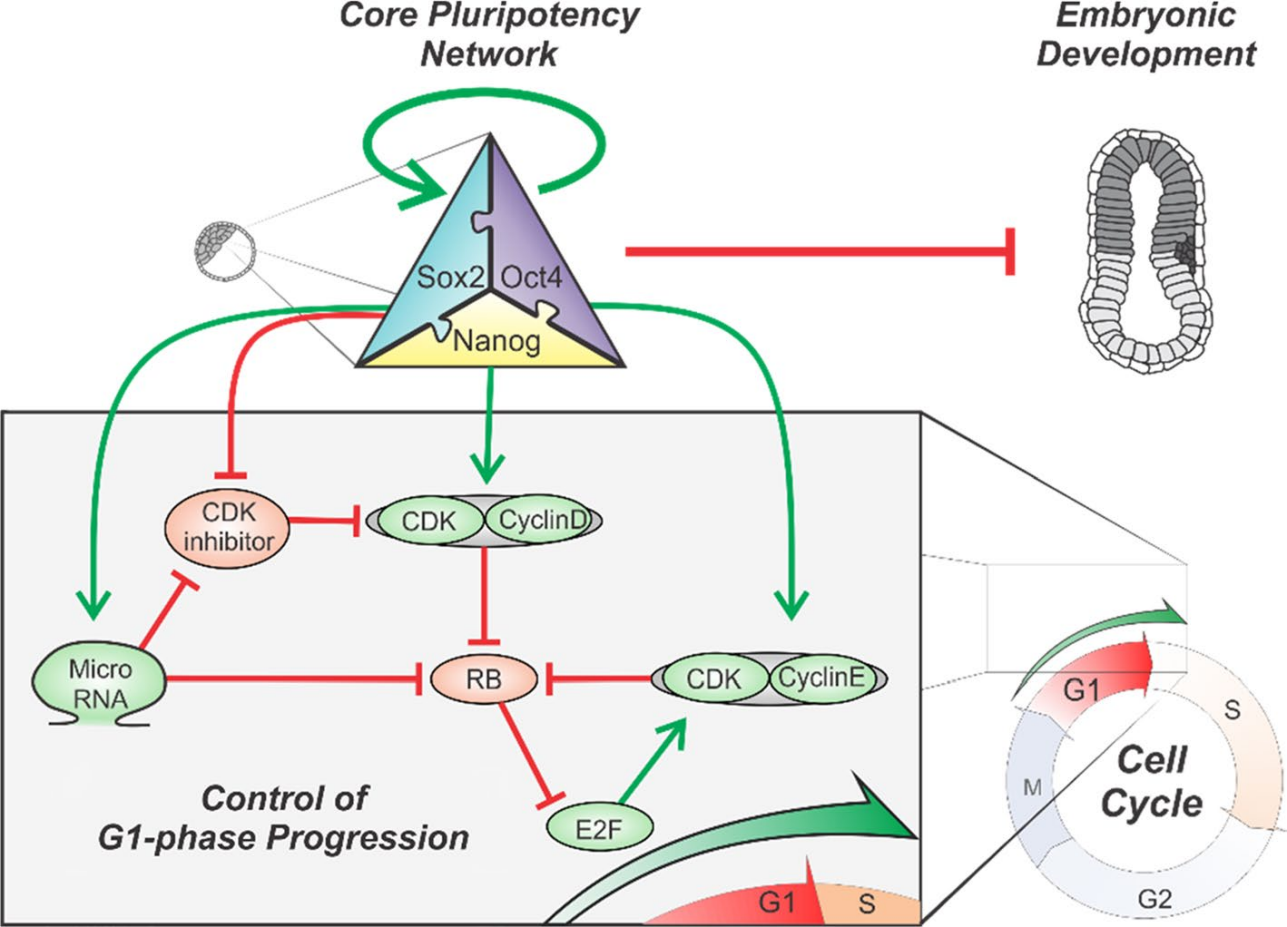
Schematic of the mechanistic link between the pluripotency network and the cell cycle regulatory network. Members of the core pluripotency network promote G1-phase progression by the activation of CDK/Cyclin complexes. In addition, transcriptional activation of microRNAs results in silencing of CDK-inhibitors and members of the pocket proteins that delay S-phase entry

Together, these results suggest that the abrogated G1-phase in naive ESCs is the result of high FGF/ERK activity and low Wnt signaling. Activation of Wnt signaling leads to transcription of the INK4/ARF locus and P16/P19-mediated elongation of G1-phase. Although inhibition of FGF/ERK induces the expression of P21 and P27 and reinstates the RB/E2F-mediated G1-checkpoint its exact molecular mechanism has not been deciphered yet. Possibly the lowered MYC expression in the ground state compared to naive ESCs contributes to the reinstatement of the G1-checkpoint (Fig. 1).

### Effect of cell cycle regulators on pluripotency and lineage specification

The discovery that somatic cells can be reprogrammed to induced pluripotent Stem (iPS) cells inspired many to combine this method with genome-wide knockdown or overexpression screens to identify genes that modulate the pluripotency network. Amongst others, many cell cycle regulators were frequently identified to improve reprogramming [60, 61]. Although reprogramming is a stochastic process that is highly susceptible to changes in cell proliferation rates [62], several studies have indicated that there are direct mechanistic links between certain cell cycle regulators and the pluripotency network (reviewed by [63]). In the following section, we will review recent work that has uncovered a direct mechanistic links between the proteins that drive G1-phase progression and the pluripotency network.

**Table:** 

Cell cycle regulator	Target	Role	References
Cyclin D1-3, E1-2	Oct4, Sox2, Nanog	Maintenance of pluripotency	[64]
Cyclin D1-3	TGFb	Endoderm specification	[15]
Cyclin D1	Endo-, meso- and ectoderm genes	Endoderm specification Neuroectoderm differentiation	[65]
Cyclin E1-2		Extra-embryonic lineage specification	[67]
CDK1	OCT4	Trophectoderm specification	[71, 72]
	ERK signalling	Mesoendoderm specification	[70]
	Epigenetic writers, Dot1L	Endoderm specification	[74]
	TFCO2L1	Maintenance of pluripotency	[75]
CDK2	Oct4, Sox2, Nanog	Maintenance of pluripotency	[64]

### CYCLINS

Although there is a general consensus that the main function of CYCLIN D and E is to drive progression through G1-phase, the cell cycle of ground state mouse ESCs does not depend on either one of them. Surprisingly, the combined depletion of Cyclin E1-2 and D1-3 does neither abrogate G1-phase progression nor successful S-phase entry in ground state ESCs [64]. Interestingly though, these cyclins elicit an important role in the maintenance of pluripotency of ground state mouse ESCs through the stabilization of proteins of the core pluripotency network, NANOG, OCT4 and SOX2. ESCs that lack both Cyclin D and Cyclin E show increased expression of Cdx2 and Eomes, two transcription factors that confer trophectodermal differentiation [64]. The ability of G1-cyclins to prevent differentiation had been observed before, although employing a different mechanism. Cyclin D1-3 and associated CDKs contribute to the maintenance of the undifferentiated state of ESCs by blocking the TGF-β signaling pathway that induces endoderm differentiation [15]. In addition, Cyclin D1 acts not only in partnership with CDKs but is also able to directly repress the expression of endoderm genes by binding and recruitment of transcriptional repressors to their promoters [65]. Together with the fact that endoderm differentiation correlates with loss of CYCLIN D1 expression and the fact that Cyclin E delays differentiation as a result of a loss of Esrrb [66] these results suggest that CYCLIN D and E are essential for upholding the core pluripotency network in epiblast cells of the ICM whereas trophectoderm cells and cells destined to contribute to the primitive endoderm lose the expression of these cyclins. In line with these results, CYCLIN E not only regulates G1 progression but impairs the differentiation towards extra-embryonic lineages as well [67]. Similarly, in the early blastocyst CYCLIN E colocalizes with NANOG in the epiblast whereas it is downregulated in trophectoderm cells that proliferate at a much slower pace [8, 68]. The fact that CYCLIN D and E knock out ESCs retain the ability to differentiate to either of the three major germ layers indicating that G1-cyclins are critical for maintenance of pluripotency but not required for germ layer specification [64].

### CDKs

Not only the cyclins but also their catalytically active partners have been functionally linked to regulation of gene expression in embryonic stem cells and during lineage specification. Similar to CYCLIN D and E the CDKs that promote progression through the G1-phase, CDK2 and CDK4, are dispensable during embryonic development until midgestation indicating that they are not required for the cell cycle of undifferentiated cells of the ICM. The loss of CDK1 on the other hand abrogates the first cell division upon conception and is considered to be the only CDK essential for undifferentiated cells to proliferate (reviewed in [69]). The fact that CDK1 expression parallels the expression of pluripotency genes and that downregulation results in differentiation underpins its importance in pluripotency [70]. CDK1 is not only essential for proliferation of ICM cells but can also interact with OCT4 and thereby promote the repression of specifiers of the trophectoderm lineage [71, 72]. Alternatively, CDK1 regulates the activity of Oct4 through a series of phosphorylation events [73]. Moreover, CDK1 is involved in a cell cycle-independent pathway that suppresses ERK signaling-mediated differentiation [70]. Together these findings suggests that CDK1 plays a crucial role in the early blastocyst that resembles the role of CYCLIN D and E. In addition, a recent study has identified several proteins involved in ESC maintenance as substrates for CDK1. CDK1 has been shown to regulate the activity of epigenetic writers and thereby prevent endodermal differentiation [74]. Similarly, CDK1 phosphorylates and thereby activates TFCP2L1, a transcription factor that prevents lineage commitment and that is essential for pluripotency in mouse ESCs [75]. Besides CDK1 an important role in maintaining pluripotency has been attributed to CDK2. Although CDK2 is dispensable in ICM cells loss of CDK2 does prolong the duration of the G1-phase [76]. In addition, CDK2-mediated phosphorylation of SOX2 enhances reprogramming MEFs [77] and de-repression of CDK2 prevents differentiation upon LIF withdrawal [78]. Not only SOX2 but also NANOG and OCT4 act as targets for CDK2-mediated phosphorylation [64]. The CDK2-mediated phosphorylation of these core pluripotency factors inhibits ubiquitination and subsequent degradation and appears to be essential for the maintenance of pluripotency. CDK-mediated phosphorylation of SOX2 also promotes the ability of a truncated form of SOX2 to negatively regulate neuroectodermal differentiation [79]. A recent study has uncovered how CDK4 and CDK6 act in concert with D cyclins to prevent TGF-β signaling-driven endoderm differentiation through phosphorylation of the SMAD proteins [15]. On the contrary, the CDK4-6/CYCLIN D activity promotes neuroectoderm differentiation. Besides the core pluripotency factors also the activity of MYC is influenced by CDK activity. CDK8 phosphorylates and protects MYC from degradation. By doing so CDK8 enhances MYC target gene expression and keeps ESCs in their pluripotent state [80]. CDK9, a less well characterized member of the CDK family binds KLF4 and contributes to Polymerase II release at the promoters of genes belonging to the pluripotency network [81]. Altogether these results indicate that the high activity of CDKs not only results in rapid cell cycle progression in ESCs, but also contributes to a transcriptional program that favors maintenance of pluripotency. However, the facts that only CDK1 is essential in the blastocyst, that most CDKs are expressed upon differentiation and that CDK-mediated modulation of the pluripotency network merely inhibits the differentiation into specific lineages suggests that CDKs are involved in lineage specification.

### CDKi

Considering the role of CDKs in ESCs it seems evident that their naturally occurring counterparts have the opposite function. This assumption is underscored by a large body of literature that indicates that CDKi’s are upregulated upon differentiation (reviewed by [82]). Apart from blocking CDK/Cyclin interactions several CDKi’s can both repress and activate gene expression by directly interacting with TFs (reviewed by [83]). The convincing evidence that CDKi’s can negatively influence the pluripotency network comes from two studies on the role of the CIP/KIP proteins. P21 is able to repress Sox2 expression by directly binding to a Sox2 enhancer [84] and P27 in complex with amongst others P130 is able to epigenetically silence the *Sox2* gene resulting in differentiation of ESCs [85–87]. Presumably, P21 and P27 bind the same repressive complex and prevent the expression of target genes during G1-phase.

Although these results imply that the expression of CDKi’s correlates with differentiation, two recent reports show that the picture is more complex and context dependent. Active Wnt signaling via TCF1 controls the expression of the Cdkn1a tumor suppressor locus, that encodes the cell cycle inhibitors P15, P16 and P19 resulting in decreased cell proliferation while leaving the expression of pluripotency genes unchanged [39]. In a recent report we have shown that ground state ESCs also express active P21 and P27 resulting in an elongated G1 phase. Despite the higher levels of P16, P21 and P27 protein, no difference in the expression of Sox2 nor other core pluripotency genes was observed [22, 88]. The fact that human ESCs also express P27 confirms that maintenance of pluripotency and the expression of CDKi’s are not mutually exclusive [16].

### RB/E2F

The general notion that arises from studies on the role of RB family proteins during embryonic development and in ESCs is that these proteins mainly play a role in differentiation. Mouse embryos lacking either Rb or both P107 and P130 die during peri-implantation stages and display hyper-proliferation and deregulated differentiation. Conform these phenotypes, in vitro cultured ESCs RB^−/−^ cells show diminished differentiation potential (reviewed by [89]). These results might be due to the fact that RB is a transcriptional repressor of Sox2 and Oct4 in somatic cells [87]. In mouse ESCs the pocket proteins are not functional and, hence, the ablation has no effect on their proliferative capacities or potential to form alkaline^+^ colonies [90]. On the other hand ground state ESCs do have a functional RB/E2F axis and the loss of RB, P107 and P130 in these cells, therefore, results in premature S-phase entry [22]. These results imply that the activity of pocket proteins might not only control G1-phase progression but negatively affect the pluripotency network in ground state ESCs as well. Despite the differential activity of these pocket proteins in naive and ground state ESCs these cells do not display major differences in the expression of core pluripotency factors [6, 88]. Furthermore, when comparing WT ESCs and ESCs lacking the pocket proteins no major differences in the expression of Oct4 were observed either [22]. Notably, pluripotent human ESCs express hypophosphorylated Rb as well, confirming that the expression of active Rb alone does not necessarily result in differentiation [90]. It remains to be determined what causes these seemingly contradictory findings. Possibly one of the other proteins of the repressive complex is not available in ground state ESCs [85, 87]. Whether the core pluripotency network of ground state ESCs is de-sensitized to Rb-mediated repression or that depletion of the pocket proteins could even amplify the pluripotency network remains elusive. Although the majority of studies have suggested that Rb promotes differentiation a recent study has identified a complex containing Rb that enhances stemness. Whether this complex is expressed and active in mouse embryonic stem cells and or during mouse early embryogenesis has not been determined [91]. Like the pocket proteins, the transcription of Sox2 gene can also be regulated by E2F transcription factors. Two closely related E2F proteins, E2F 3A and 3B, have opposing effects on the expression of Sox2. Both transcription factors bind within close distance of the Sox2 Transcription Start Site (TSS) but E2F3B results in lowered deposition of H3K27me3 whereas replacement of E2F3B with E2F3A resulted in the recruitment of a repressor complex [92]. How they exactly contribute to the regulation of the pluripotency network is, however, unclear. The combined deletion of all three “activator” E2Fs, E2F1-3, did not abolish self-renewal of naive ESCs nor efficient teratoma-formation [93]. Only a few specific tissues showed impaired proliferation and increased apoptosis suggesting that they might contribute to differentiation. In agreement with these studies, it had been shown that none of the studied E2F family members is essential for cells of the ICM [94].

### P53

If cells suffer from stressful conditions progression through the cell cycle can be temporarily halted during G1-phase. The pathways that are responsible for G1-arrest not only act in response to stress but have also been shown to mediate developmentally programmed cell senescence [95]. P53is an important tumor suppressor and master regulator of stem cell quiescence [96]. Although highly expressed and active at early embryonic stages [97, 98] P53^−/−^ mice are viable and a debate on the role of P53 in embryonic development is still ongoing [99]. A large number of studies have indicated that P53 in naive ESCs is transcriptionally inactive under normal circumstances but can mediate differentiation under stressful conditions by both inducing the expression of lineage specifiers and downregulation of the core pluripotency network (reviewed by [100]). Upon DNA damage, for example, P53 binds to the Nanog promoter and reduces the expression of NANOG [101]. Moreover, it can repress OCT4, NANOG, and SOX2 by interfering with distal enhancer activity [102]. Moreover, microRNAs induced by P53 target OCT4 and SOX2 [103, 104] and facilitate extraembryonic endoderm specification [105]. Recently two studies have highlighted the role of P53 in mesendoderm differentiation. P53 inhibits a transcriptional network that promotes pluripotency through the expression of long non-coding RNAs resulting in differentiation into the mesoderm and endoderm [106]. These results were confirmed by the finding that the combined deletion of P53, P63 and P73 resulted in abrogated mesendodermal differentiation. The P53 family tweaks the collaborative WNT/TGF-β pathway by inducing the expression of several WNT ligands [107].

**Fig. 2:**
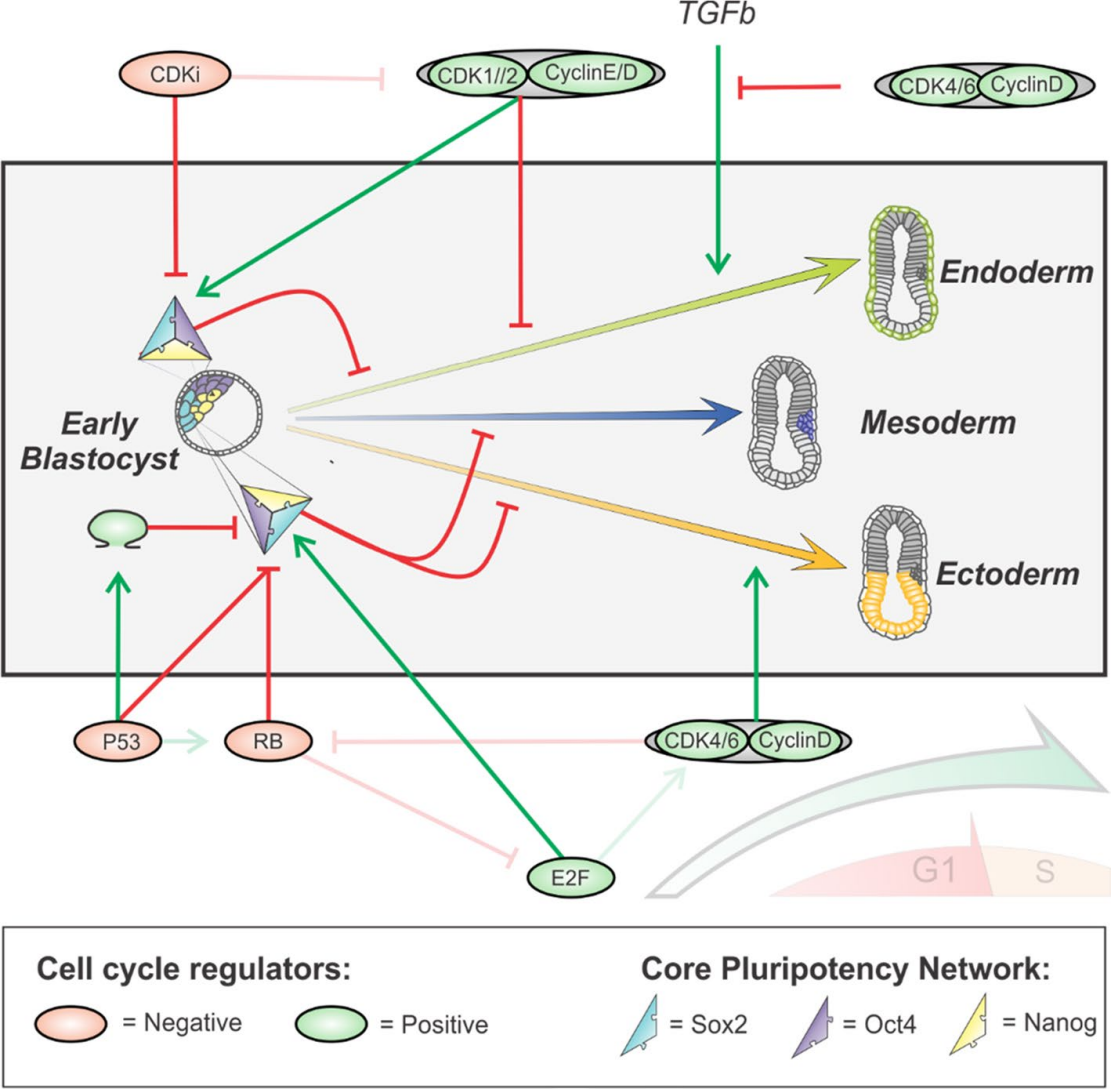
Schematic overview of how canonical cell cycle regulators affect the core pluripotency network and lineage specification

In contrast to the above-mentioned literature, a few studies have shown that P53 can also inhibit lineage commitment and contribute to an undifferentiated phenotype. Stress-induced P53 activity was shown to mediate the transcription of Wnt ligands that contribute to an undifferentiated phenotype [108]. Moreover, P53 can elevate TGF-β signaling and concomitantly NANOG expression prevent the exit of the pluripotent state by elevating TGF-beta signaling and accompanying NANOG expression [109] (Fig. 2).

## Discussion

**Fig. 3:**
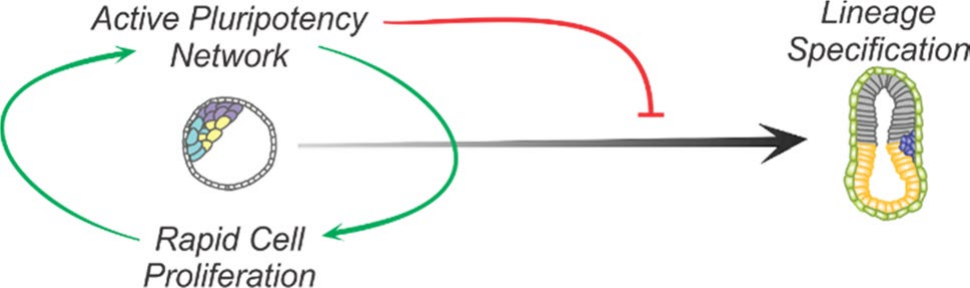
Proposed model of interdependence of the pluripotency network and the cell cycle

Early studies on ESC derived from the inner cell mass revealed an unusual short cell cycle structure that was subject to dramatic changes during cell fate specification [25, 110]. The cell cycle regulated differentiation and accompanied chromatin remodeling led scientist to believe that the ESC-specific cell cycle structure and differentiation are mechanistically linked. Whether the ESC-specific transcriptional program that coordinates pluripotency also facilitates their highly proliferative phenotype and characteristic short G1-phase has been a subject of debate [63]. In this review, we have summarized the molecular mechanisms driven by signaling pathways that dictate early embryonic development and the core pluripotency TF machinery plus MYC that impact on G1-phase progression. In addition, we have tried to rationalize to what extent these biological processes explain the distinct cell cycle structures of ESCs in different states of pluripotency [6, 111]. Although ESCs in both states proliferate at high rates, signaling pathways that ensure maintenance of pluripotency do not confer unrestricted G1-phase progression, the relative short G1-phase is not a feature of ground state ESCs [22, 40]. In line with these observation, the presence of hypo- but not hyper-phosphorylated pocket proteins in ground state ESCs suggest that pluripotency factor-driven CDK/Cyclin activity is not characteristic of ground state ESCs and therefore not an intrinsic feature of pluripotent ESCs. Together these results imply that fast G1-phase progression and cell proliferation is not driven by pluripotency. Furthermore, we have discussed how members of the canonical G1-restriction point modulate pluripotency and differentiation. In general, pro-proliferative members, like cyclins and CDKs, have a positive effect on the maintenance of pluripotency. Conversely, the activity of members that delay G1-phase progression correlates with differentiation [69]. Many members that promote proliferation and pluripotency are, however, dispensable in the early ICM and rather prevent differentiation than promote pluripotency [15, 64]. The latter can be explained by the fact that the initial phase of tissue development not only requires proper cell differentiation but sufficient cell numbers (Fig. 3).

### Future perspectives

Over the past decades, the unusual short G1-phase of in vitro cultured ESCs has been the subject of intense investigation. Although this has led to an increasing understanding of how pluripotency and the cell cycle are intertwined, the cell cycle dynamics, and in particular the duration of the G1-phase, during embryonic development in vivo has remained largely unexplored. As outlined above, the short G1-phase of in vitro cultured ESCs is driven by several signaling pathways that play pivotal roles in embryonic development. Whether these signaling pathways responsible for the rapid increase in cell number during the cleavage stage as well, and whether this is through shortening of the G1-phase, is yet to be determined. Future studies combining genetic engineering and live cell cycle reporters could address these fundamental questions in developmental biology.

Besides the role of the shortened G1-phase during in vivo embryonic development the effect of the extremely short G1-phase on the genomic integrity of in vitro cultured ESCs remains another interesting avenue for future research. Several studies have indicated that the short G1-phase in ESCs cultured in serum-rich conditions compromises the G1-specific DNA damage checkpoints. The absence of such checkpoints may in turn affect the genomic integrity of in vitro cultured ESCs. Whether the abbreviated G1-phase impairs DNA damage repair in ESCs in vitro, and whether this results in the elevated accumulation of DNA damage is not entirely clear. In this light it would be interesting to assess whether ESCs grown in serum-free conditions, that have an elongated G1-phase, cope differently with DNA damage during G1-phase. These studies will shed light on how different culture conditions affect the accumulation of DNA damage and the genomic integrity of ESCs. The results might have important implications for the clinical application of ESCs, such as therapies aimed at using stem cells-derived cells to replace damaged tissue.
